# Seismic Performance of Recycled Aggregate Geopolymer Concrete-Filled Double Skin Tubular Columns with Internal Steel and External FRP Tube

**DOI:** 10.3390/polym14235204

**Published:** 2022-11-29

**Authors:** Yasser Alashker, Ali Raza

**Affiliations:** 1 Department of Civil Engineering, College of Engineering, King Khalid University, P.O. Box 394, Abha 61411, Saudi Arabia; 2 Structural Engineering Department, Faculty of Engineering, Zagazig University, Zagazig 44519, Egypt; 3 Department of Civil Engineering, University of Engineering and Technology Taxila, Taxila 47050, Pakistan

**Keywords:** recycled aggregates, glass-FRP tube, seismic behavior, geopolymer, quasi-static load

## Abstract

The large production of cement is resulting in a high-carbon footprint, which is essential to minimize for sustainable concrete construction. Moreover, the large quantity of recycled coarse aggregate (RCA) from the demolition of old concrete structures is creating problems for landfill and disposal. The primary goal of this study is to investigate the seismic efficiency of innovative fiber-reinforced polymer (FRP)-recycled aggregate geopolymer concrete (RAGC) steel-tubed columns (FGSTCs) with an internal steel tube (STT), an external glass-FRP tube (GLT), and RAGC located between the two-tubed components to develop a serviceable structural element. To study their seismic functioning under axial load and lateral repeated load, five FGSTC specimens were manufactured and analyzed under quasi-static loads. The influence of three variables on the performance of FGSTC specimens, consisting of STT reinforcing ratio, compression ratio, and recycled coarse aggregate (RCA) replacement ratio, was investigated in this investigation. The produced specimens’ ductility, hysteretic loops, strain distribution, skeleton curves, stiffness functioning, energy capacity dissipation, damaging functioning, and strength loss were all assessed and discussed. The results of this investigation revealed that percentage substitution of RCA had a minor impact on the seismic functioning of FGSTCs; however, the compression-load ratio depicted a substantial impact. The energy loss of the FGSTCs was 24.5% higher than that of their natural aggregate equivalents. FGSTCs may have a 16.9% lower cumulative failure rate than their natural aggregate counterparts.

## 1. Introduction

The expanding global population, massive urbanization, and the world’s current economic situation have all contributed to an amelioration in the use of recycled coarse aggregate (RCA) concrete made from the development and demolition of buildings. The application of RCA in concrete has been discovered as a good technique to create a cost-effective, environmentally friendly, and long-lasting building. The RCA offers the advantages of shortening aggregate shipping distances and minimizing the amount of landfill area required for building and demolition debris. It is also environmentally friendly, releasing very little carbon [1,2,3,4,5]. When compared with conventional aggregate concrete (NAC), the concrete made with RCA has flaws such as lower compression strength, higher water holding capacity, and higher porosity. RCA concrete, on the other hand, was found to be more ductile than NAC concrete [6]. Nevertheless, just a few studies have shown that incorporating recycled aggregate concrete (RAC) into concrete manufacturing is effective. The manufacture of Portland cement releases a substantial quantity of carbon dioxide (CO_2_). The use of RCA in geopolymer concrete (GPC) is expected to minimize cement and concrete production’s high carbon footprint. In GPC concrete, alkali activators such as fly ash, silica fume, blast furnace slag, and red mud produce inorganic alumino-silicate polymers. While there is a variety of literature on this topic about the mechanical and durability properties of both RCA and GPC elements independently [7,8,9,10,11], no research has been conducted on the simultaneous use of GPC-RAC columns restricted with fiber-reinforced polymers (FRPs) and steel tube.

Several experiments have produced positive results by externally strengthening concrete columns using fiber-reinforced polymers (FRPs) over the previous few decades [12,13,14,15,16,17,18,19]. Due to their good properties, specifically a high strength-to-weight ratio and beautiful corrosion resistance, FRPs have experienced incredible ubiquity in strengthening the unique structural specimens due to their good properties [20,21,22,23]. The limitations of FRPs, which work on both deformities and strength implementation of the concrete, can be used to control the lateral expansion of concrete under axial strength [24,25]. The use of FRPs in conjunction with RAGC will result in a structural element that is both lightweight and ecologically sustainable. In addition to higher strength-to-weight ratio and beautiful corrosion resistance, the fatigue and creep resistances are critical when considering the cyclic loading. These characteristics of FRPs, especially carbon-FRPs, are good enough to bear aggressive environments and loading conditions such as dynamics and static loadings [26,27,28]. Hadi et al. [29] studied the structural performance of GPC columns reinforced with basalt-fiber-reinforced-polymer (BFRP) bars and/or restricted with BFRP tubes. When compared to columns made of regular Portland cement (OPC) concrete, they found that the steel-reinforced GPC would have 7% greater axial compression strengths (ACS) but 11% lower ductility. Furthermore, confining GPC columns with BFRP bars and tubes resulted in ACS and ductility that were 14% and 3% higher, correspondingly, than OPC columns. Danda et al. [30] used ground granulated blast furnace slag to investigate the influence of sodium hydroxide (NaOH) molar concentration on the ACS of reinforced GPC columns (GGBS). According to their findings, the LCC and axial deformation improved as the NaOH concentration was ameliorated. Both curing temperatures and curing duration have a significant influence on the performance of GPC columns [31]. Saranya et al. [32] reported that under compression load, steel fiber-reinforced dolomite-GGBS GPC short columns have a greater ACS than steel fiber-reinforced OPC columns. Maranan et al. examined the structural performance of glass-FRP-reinforced GPC columns and found that their compression performance was far better than that of their OPC equivalents [33]. When compared to hoop-confined GPC columns, spiral-confined GPC columns had significantly higher confinement efficiency and ductility. The GPC columns reinforced with glass-FRP bars exhibited good structural functioning in these investigations. More research should be conducted to establish the structural functioning of glass-FRP reinforced GPC utilizing RCA for compression elements.

Past research has shown that concrete subjected to the demands of both FRP tubes and steel tubes (STT) has a remarkable ductile functioning that is vital and eye-catching in seismic locations [34,35,36,37]. FRP-concrete-steel double-skin tube confined columns evaluate all of the components of such composite sections, which comprise two tubes and inner concrete [38,39,40]. Because of the RAGC-filled space between the two tubes, an effective FRP-RAGC-STT column (FGSTC) with an applied external glass-FRP tube (GLT) and an inside-placed STT will work more effectively under earthquake load. In comparison to natural aggregate concrete, fiber-reinforced steel tube columns not only take advantage of double-skin tube confined columns in terms of reducing the incompetence of recycled materials in these elements but also make them extra lightweight and improve the ductility implementation of recycled aggregates [41]. This suggests that the newly fabricated FGSTC column structure has a lot of potential for practical use in earthquake zones. Internal STT might help with problematic incomplete compressive strength and ameliorate the bending stiffness of elements to compensate for the insufficiency of interior filled concrete, and the empty center composite reduces the weight of the column [42]. Muhammad Akhsin and Fiedler [43] investigated the failure prediction and surface characterization of glass FRP (GFRP) laminates. In this work, stepwise loading was used to analyze how multiple static loads affected the laminates before final failure. With loading-unloading motions before the last load until failure, the loading was set three times to reach 10 kN. The outcomes demonstrated that a critical factor in the system’s failure is the angle at which the GFRP UD laminates are positioned. The [0/90]S laminates, which have a 0° layer on the edge, first experienced preliminary failure.

In any case, research into the seismic execution of FGSTCs is severely limited, as seen by the difficulties of conducting quasi-static compression testing on compression specimens [44,45,46,47]. Because the earth is going through an earthquake damage phase, seismic activity has occurred often for many years, resulting in remarkable tragedies such as near-miss injuries and structural demolition of buildings in a short period [48,49]. Critical instability of concrete structures was mostly caused by damage to elastic columns, which was primarily caused by a faulty cross-over limitation [50]. As a result, it is critical to evaluate FGSTC seismic functioning before they are widely used in actual applications. Furthermore, because FGSTCs are expected to have ameliorated ductility, it is energetic and logical to reuse obliterated concrete caused by earthquakes in FGSTCs that have exceptional seismic performance. The next point of worry is that there have not been any further investigations employing full-scale FRP-confined elements. Applying the results of limited scope specimens directly into the pragmatic design may result in information perforation, whereas the sampling element has a substantial impact on its energy dissipation capability [51]. During the glass-FRP assembly loop, it is acknowledged to be a lesser failure to the ecosystem [52]. As a result, this sort of FRP was chosen for use in this study because it seems to have a promising future for practical use. Furthermore, two pre-assembled FRP tubes were employed as formwork for RAGC projecting in the construction activities without expulsion, resulting in less carbon emissions [53].

This research aimed to offer ecologically acceptable structural components using the RAGC, as well as to ameliorate the practical uses of reusable RAGC in earthquake zones for sustainable development. The primary goal of this research is to look into the seismic functioning of five FGSTCs that were put through quasi-static study under compressive load and seismic lateral pressures. During the construction of the columns, three factors are taken into consideration: recycled coarse aggregate substitution, axial compression loads ratio, and reinforcing ratios. Axial load capacity, deformation efficiency, destructive pattern, and energy emissions were all assessed parameters of the tested columns. The positive findings of this study will aid structural engineers in designing durable, lightweight, and sustainable concrete columns.

## 2. Materials and Method

### 2.1. Materials

#### 2.1.1. Recycled Aggregate Geopolymer Concrete

**Figure 1:**
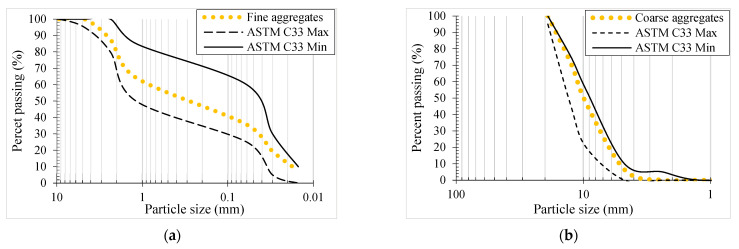
Granulometry (**a**) fine aggregates (**b**) RCA (C33 indicates the ASTM code, C33 Max and C33 Min indicate the maximum and minimum allowed passing percentages from the specific standard sieves).

The coarse aggregates were replaced with RCA to create the RAGC. In the RCA, concrete specimens with compression strengths varying from 25 MPa to 50 MPa and durations lasting from 6 to 12 months were used. The parameters of recycled aggregate are shown in Table 1. The maximum size of the recycled aggregate was 12 mm. For the mixing process, local Lawrancepur sand was employed. Figure 1 shows the granularity characterization of the RCA and the sand. A Sika ViscoCrete^®^-3425 superplasticizer (provided by Sika Pakistan) (20 kg/m^3^) was incorporated into the GPC blend to verify that it worked properly. The GPC mix with a nominal density of 2500 kg/m^3^ was produced using a trial-based testing procedure. In other words, the required strength and mix design of GPC were obtained by performing some initial tests. Class F fly ash (45%) and GGBS (55%) were employed as binders, both of which are available on the market as waste products. A combination of sodium hydroxide with a molar concentration of 14 M and Na_2_SiO_3_ with a mass ratio of 1:2.5 was utilized for stimulation. As determined by ASTM C143/C143M-15 [54], the workability of fresh RAGC was 125 mm. As per ASTM C807-13 [55], GPC had a set time of 90 min. To test the RAGC’s compression strength, three concrete specimens sized 150 mm × 300 mm and three concrete cubes sized 100 mm × 100 mm were poured from each mix proportion. The cylinders and columns were both put to the test on the very same day. The mix designs of the RAGC with varied RCA weight fractions are shown in Table 2.

#### 2.1.2. Steel Tube

In this study, ultimate tensile strength, yielding strain, yield strength, elongation, Young’s modulus, and fracture modulus are among the many STT characteristics used in this study and are listed in Table 3. The widths of the STT were chosen based on the factors that the width of the STT can be large in order to have a large second moment of area to resist the moment, while the empty portion should not be sufficient to consider with enough concrete, which plays a vital role in carrying loads.

#### 2.1.3. Glass-FRP Tube

For this work, GLT with fiber orientations at 80^o^ from the axial direction of the specimens was used for this work to provide a suitable transverse confining method to the core concrete and outside STT. FRP tubes were taken from SupAnchor^®^ (Dujiangyan, Sichuan, China) GLT had a diameter of 400 mm and a thickness of 4 mm. The ultimate strength and elastic modulus of GLT secured from tensile tests were 650 MPa and 50 GPa, correspondingly.

### 2.2. Manufacturing of Specimens

**Figure 2:**
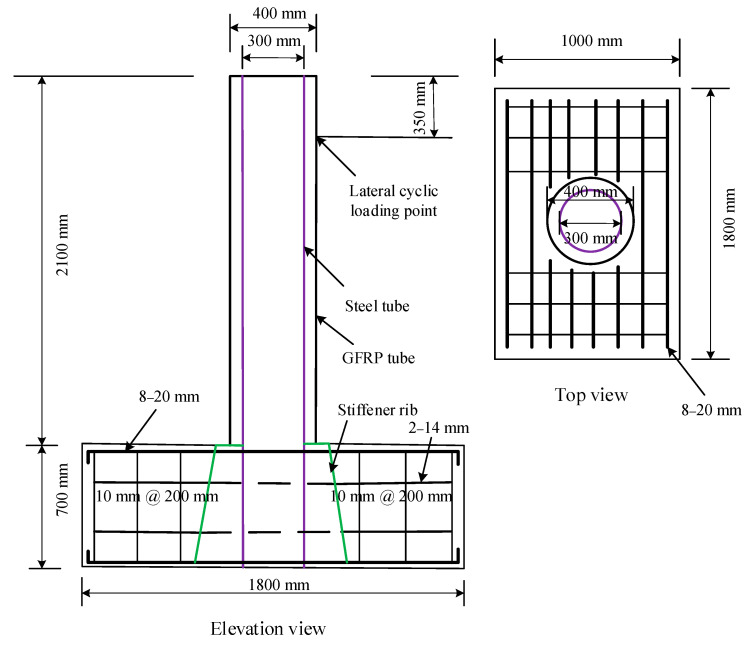
Elevation and plan views of FGSTC specimens.

The specimens used in this study were made to form a semi-column with a cantilever at one end and a fixed end. Hand layup procedure was used to fabricate the test specimens. The quasi-static load was applied to the cantilevered end of the specimens. A base was mounted to the base of the column section to examine the seismic effect, while the upper end of the column was employed to give both cyclic and axial load. Under quasi-static stresses, five FGSTC specimens with circular glass-FRP and STT were examined. The specimens were encased in GLT with an internal diameter of 400 mm and a wall thickness of 4 mm internally. The STT was used with a 300 mm wide and an 8 mm or the 10 mm plate thickness. The specimens had a height of 2800 mm, with 700 mm for the base and 350 mm for the apparatus. The useful height of FGSTC columns for testing was 1750 mm, measured from the point of lateral load to the top section of the base footing. The footing was 1000 mm × 1800 mm in size when viewed from the upper side. The geometric model and elevation views of the FGSTC specimens are shown in Figure 2.

The three parameters studied were the axial load ratio (0.4 and 0.2), the percentage of recycled aggregates (r = 0 percent, 50%, and 100%), and the reinforcing ratio of composite specimens (3.04 percent and 3.81 percent). The volumetric quantity of coarse aggregates was used to substitute them. In this work, the axial load ratio was defined as the ratio of axial force to crushing load. The reinforcing ratio was calculated as the ratio of the area of STT to the overall area of the specimen when the thickness of STT was changed. The slenderness ratio of specimens was also significantly affected by the STT reinforcing ratio. The geometrical parameters of the specimens are shown in Table 4.

### 2.3. Testing Preparation

Every specimen was prepared with an FGSTC in which the STT base column was put into a solid RC balance. Firstly, welding was used to attach eight stiffeners made of steel and two steel plates to the STT base end, and then primary reinforcement rebars were joined to the steel plates. Additionally, after incorporating strain measures near the column end of the STT, ordinary concrete was expected to be in the balance. The strain gauges should have been sealed to insulate them from steel and humidity when the STT was constructed. In the STT, a GLT was set and constrained. Between the tubes, RAGC was developed. The upper part of a specimen was smoothed out with gypsum before the application of load to ensure an even-strength application on the specimen.

### 2.4. Load Mechanism of Specimens

**Figure 3:**
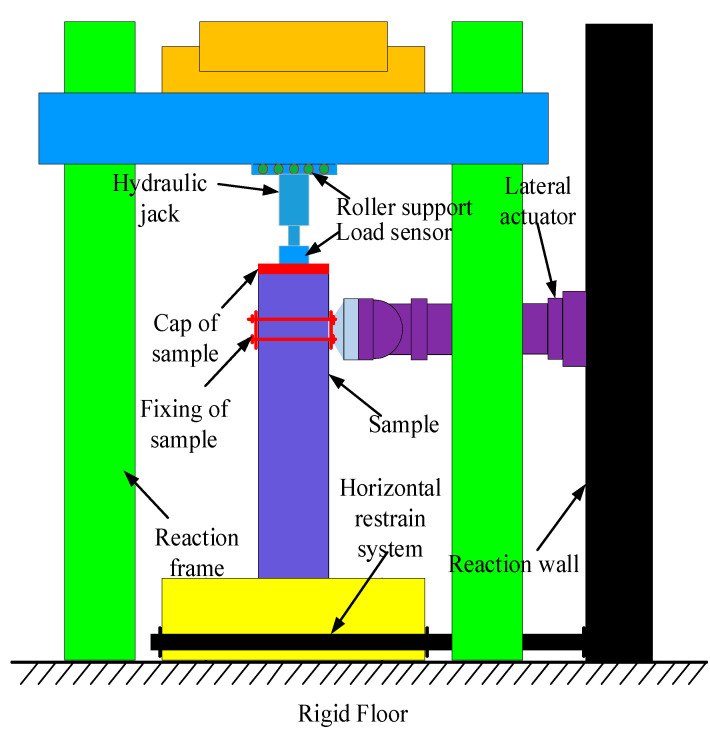
Testing stet-up for load process schematic diagram of testing arrangement.

The test apparatus, sampling, and analysis setup under quasi-static loads are shown in Figure 3. The RC balance used four steel bolts to secure the specimen’s base surface from the bottom. The compression performance was provided by a reaction frame with the highest ACS of 5000 kN in an upward position. The compression load was placed on the top surface of the specimen by a jack with the highest ACS of 3500 kN. The jack was mechanically structured, and a compression sensor beneath the load jack was linked to a data logger (TDS530, Tokyo Measuring Instruments Laboratory Co., Ltd., Tokyo, Japan) so that the compression could be monitored continuously. The load jack and the efficiency frame are connected by a movable assist, which allows the specimen head to move along the side while maintaining uniform strength. In addition, a column cap was placed on the specimen’s uppermost point under the sensor to ensure that strength was transferred evenly from the load jack to the specimen. A massive efficiency divider on a level plane provided horizontal efficiency. After the strength of concrete was set to the desired value, a horizontally positioned actuator of the load apparatus was used to apply transverse load with a tensile capacity of 2000 kN and a compression threshold of 3000 kN (Suzhou Zhubang Measurement and Control Technology Co., Ltd., Beijing, China). The specimen head was connected to the horizontally positioned actuator via a specially designed installation that accepted the column. The data was recorded using a data logger attached to a computer.

**Figure 4:**
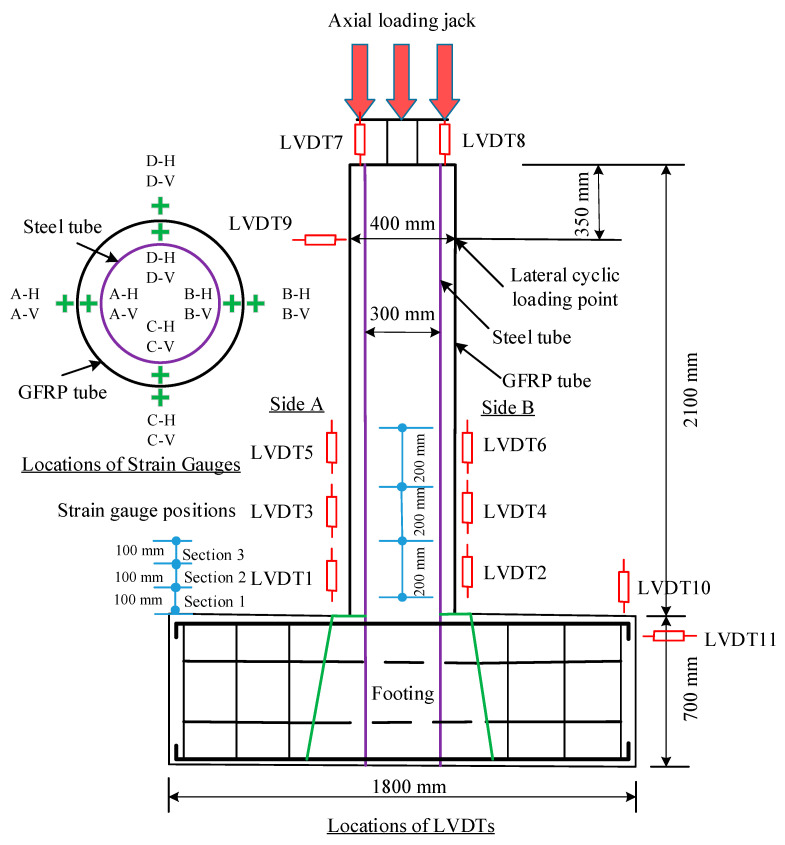
Instrumentation for LVDTs and strain gauge arrangement.

In this investigation, 11 LVDTs and 48 strain gauges were linked to every specimen and measured the specimen’s strains and deformation, as shown in Figure 4. For clarity in discussions, the transverse actuator’s pushing and pulling orientations are denoted as “positive” and “negative”, correspondingly. Both faces (B and A) of the specimens are designated as compression and tension face, correspondingly, by pushing the specimen. The side between the two sides was designated as face D. The LVDTs were 200 mm long. As illustrated in Figure 4, The LVDT1 was attached to the specimen’s face A, whereas the LVDT2 was attached to the specimen’s face B. Similarly, at 200 mm breaks, LVDTs 3, 4, 5, and 6 were attached to the specimen above the sites of LVDT1 and LVDT2. LVDT7 and LVDT8 were used to measure the compression length of the specimen at the top end. On side A, where lateral force was given, the LVDT9 was positioned in the horizontal position. Then, the two additional LVDTs were linked to the specimen’s base footing in the flat and vertical directions, correspondingly, to monitor the footing’s flat and vertical motions.

A total of four sets were formed for a total of 24 strain gauges with a length of 20 mm on GLT in terms of strain gauge layout. The STT was used to interconnect the remaining 24 strain gauges. Three suitable locations for attaching the strain gauges to GLT were identified at heights of 100 mm, 200 mm, and 300 mm from the upper portion of the base structure. Likewise, when mounting the strain gauges on the STT at elevations of 100 mm, 200 mm, and 300 mm from the upper portion of the base structure, the matching position, as in the glass-FRP rube, was taken into consideration.

**Figure 5:**
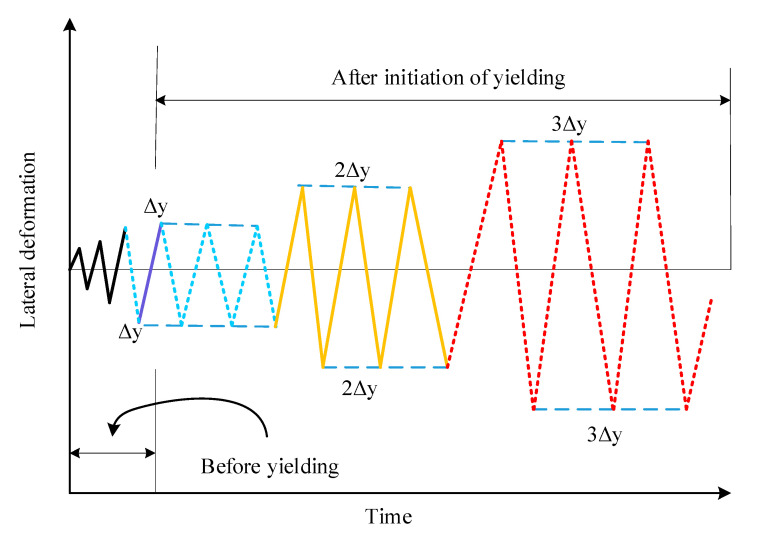
The cyclic load technique.

In this investigation, the compression load was performed employing the load-controlling approach at one kN/s. This load speed was enhanced until the desired compression strength was achieved, after which it was maintained constant throughout the experiment. The specimens are subjected to lateral dynamic load by following the displacement control techniques shown in Figure 5. After that, the displacement ameliorates of the cyclic load was considered equivalent to the yielding displacement (as represented by the nonlinear graph). The cycling procedure was executed three times. This dynamic load approach was sustained until the load was reduced to 85% of the specimen’s maximum strength. The visible fissures were visible at this point, and the specimen’s deformations had grown too great to withstand the imposed loads.

## 3. Results and Discussion

### 3.1. Failure of Specimens

**Figure 6:**
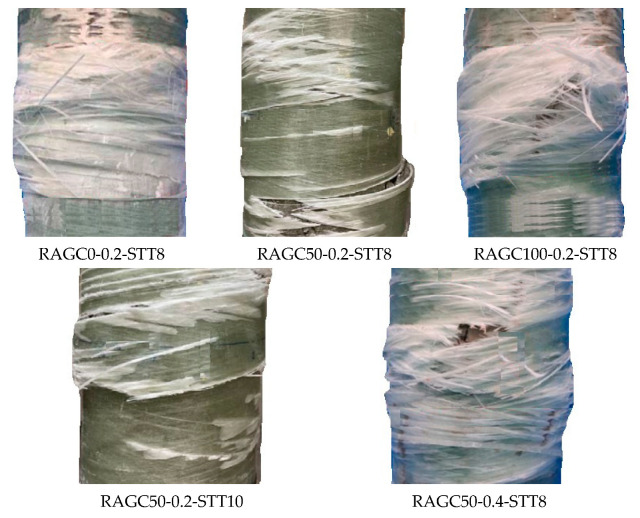
Failure modes of tested samples.

Circumferential fractures in the GLT cracks in the STT, and flattened RAGC were all common failure and cracking patterns in all of the specimens. The three phases of the damage interaction are the flexible phase, the crack development phase, and the destructive phase. The transverse deformation of each specimen was initially mild, comparable to the stresses from both tubes resulting from the application of loads (GLT and STT). The strains in both tubes (GLT and STT) continuously ameliorated as the transverse load ameliorated, accompanied by infrequent tiny breaking sounds. Soon after, the specimen’s horizontal resistance was no longer elastic, with constant increments indicating the specimen had progressed to enhance the fracture phase. Slight circumferential fractures appeared on the outside of the GLT, accompanied by loud breaking sounds. When the lateral diversion reached around 2δy, the major obvious fractures in the GLT were identified. The strands’ bending sites corresponded to the observed points of substantial cracks in the GLT, demonstrating that the tar framework fractured before the threads. As the lateral stress ameliorated, the fractures accumulated at the column’s foundation, and a few severe fractures with a total width of 3 to 5 mm developed on both sides in an area larger at 150 mm and ending at 350 mm from specimen end (B and A). The threads that are obstructing the resin incorporation at this time refuse to cooperate and easily break. Surprisingly, before the threads shattered, the points of several cracks around them shifted to the level course, implying that the thread’s stiffness had been fully utilized. The fractures on the elastic face expanded very quickly, and some substantial fractures of identical magnitude on both sides of the specimen (B and A) cooperated to accelerate damage progression. The RAGC within had been squished and was now exposed. Before reaching the limit, the application of loads improved steadily. As a result of the accumulating fractures on the GLT and the RAGC, the specimen’s longitudinal axial load capacity began to deteriorate progressively, and the specimen entered the damage phase. The GLT fibers were more shattered during this period, and the core concrete crushing was more obvious. Figure 6 shows the failure modes of some samples.

### 3.2. Skeleton Curves

**Figure 7:**
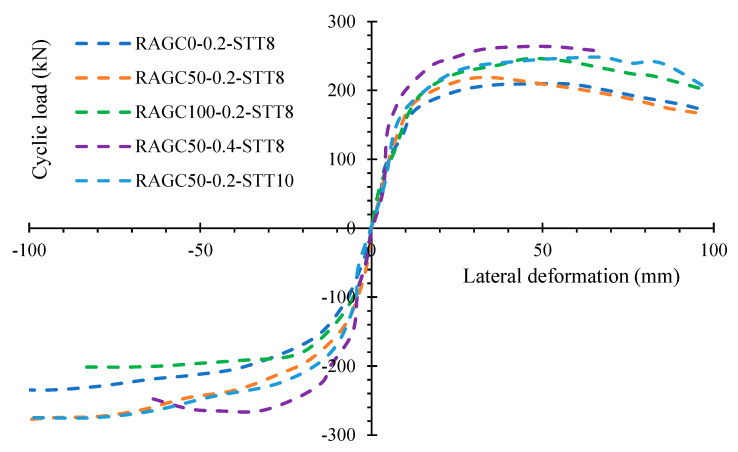
Skeleton curves.

For the most part, every specimen in these graphs had a first increasing zone and a second, larger dropping region, as seen in Figure 7. In terms of the RAGC replacement rate’s influence on specimen stiffness, three skeleton graphs largely agreed earlier in the specimen yielding, suggesting that the RAGC replacement rate had a minor impact. After the specimen was yielded, the graphs began to deviate. With an amelioration in the RCA substitution rate on face A of the specimens, the axial load capacity ameliorated. In comparison to the specimens RAGC50-0.2-STT8 and RAGC0.99-ST8, the specimen RAGC100-0.2-STT8 had an ultimate strength of 14% and 17%, correspondingly. On face A, the lateral axial load capacity decreased gradually after reaching the top, whereas on the negative side, there was no evident decay area. Specimen RAGC50-0.2-STT8 had a maximum horizontal deformation similar to specimen RAGC0-0.2-STT8, and both were larger than specimen RAGC100-0.2-STT8 was. When the influence of the compression load proportions was investigated, it was discovered that the specimens’ elastic stiffness was more noticeable at the higher compression load percentage. On face A (positive side), the skeleton profile of specimen RAGC50-0.2-STT8 was usually lower than that of specimen RAGC50-0.4-STT8, and specimen RAGC50-0.4-STT8’s axial load-carrying strength was 31 percent higher than that of specimen RAGC50-0.2-STT8. The skeleton graph of specimen RAGC50-0.4-STT8 has fewer columns on both the negative and positive sides, but mainly in the positive direction. The greatest parallel deformation of specimen RAGC50-0.4-STT8 was significantly lower than that of specimen RAGC50-0.2-STT8, indicating that specimen ductility decreases as compression proportion ameliorates. The ratio of STT had a reduced influence on specimen elastic stiffness; although the skeleton graphs showed slow deviation once, the specimens reached the plasticity phase. Specimen RAGC50-0.2-STT10 had an axial load-carrying capacity that was 8% higher than that of specimen RAGC50-0.2-STT8. Furthermore, the axial load capacity of specimens with larger steel ratios deteriorated at a slower rate than those with a small STT ratio. The RAGC50-0.2-STT8 and RAGC50-0.2-STT10 specimens demonstrated excellent ductility and a 95.5 mm severe horizontal deformation.

The compression load fraction largely affects the specimens’ axial load capacity and deformations attributes. In these experiments, the steel reinforcing proportion affected the axial load-carrying capacity but had a smaller effect on the deformation execution. The RCA substitution rate had the greatest impact on the material properties of the negative side as associated with the positive side.

### 3.3. Hysteretic Loops

**Figure 8:**
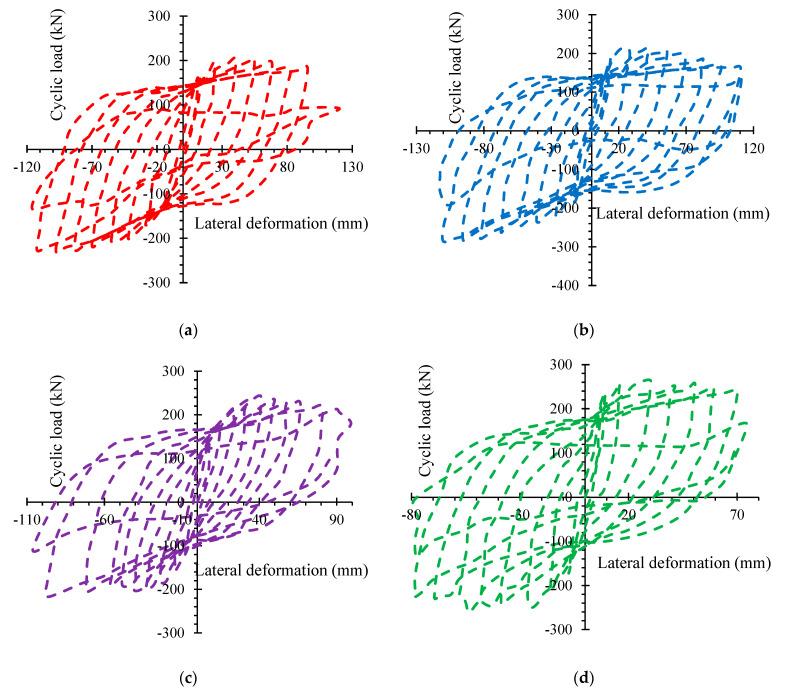

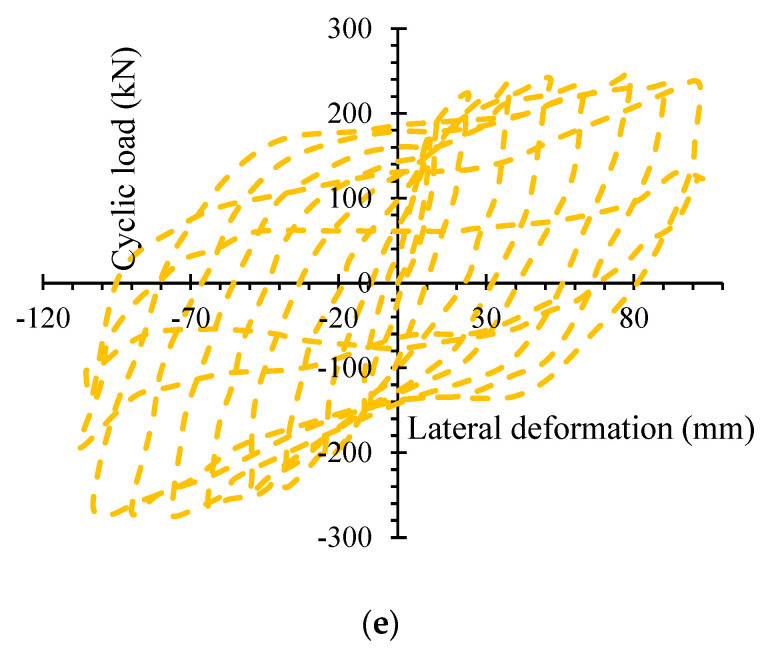
Transverse load-deformation curves of specimens: (**a**) RAGC0-0.2-STT8 (**b**) RAGC50-0.2-STT8 (**c**) RAGC100-0.2-STT8 (**d**) RAGC50-0.4-STT8 (**e**) RAGC50-0.2-STT10.

Figure 8 depicts the lateral load-lateral deformation graphs for all examined specimens. Three times, the cycling procedure was executed. The load was reduced to 85% of the specimen’s maximum limit using this cyclic load method. The visible fissures were visible at this point, and the specimen’s deformations had grown too great to withstand the imposed loads. The testing materials in this investigation were initially exposed to axial compression stress before being subjected to lateral cyclic load. The longitudinal load restriction is lessened when the specimen bends towards one section. When the specimen bends towards the other portion, the parallel load limit is ameliorated. This is transcending because the items in the specimen had failed in previous load rounds. The minor fractures of the specimen were gathered at a specific place by advancing the transverse loads, resulting in inconsistent damaged reductions for sides B and A due to non-homogeneity of components and varying local buckling functioning of the STT [56,57]. As a result, the specimen’s strains developed irregularly on these different sides. Plastic distortion performance and energy limitation dissipation were unsurpassed in fully adjusted hysteretic loops. The specimens had essentially no residual deformation before the STT began to give, and the hysteretic loops revealed decreased gaps across the transfer of axial and lateral cyclic stress. The elastic correlation can be used to represent the specimen deformation in this period. The energy dissipation of the specimens was neither apparent nor predictive of a reduction in loading capability. When the STT was yielded, growing compression caused residual stresses and gaps in the hysteretic loops to enlarge, although the load inclines or dumping curves decreased. At the initial load loop (earlier the horizontal deformation had reached 7δy), three hysteretic loops for each of the load fix loops were generally covered. The axial load-carrying capacity and stiffness peaked for transverse deformations between 4δy and 6δy. Because of the STT yielding or buckling failures, most specimens broke at lateral deformations of roughly 8–10δy. The three test variables have an impact on hysteretic functioning. As the RAGC replacement rate ameliorated, the maximum transverse deformation of the specimens dropped slightly. However, after attaining its maximum level, the horizontal load-carrying strength decreased even more slowly and without any abrupt reduction. The specimens with higher RAGC replacement rates had more compression, which could be due to the strength of RAGC. By raising the axial load fraction, the graph’s P-impact is maintained better with a greater compression load, resulting in extended longitudinal deformation and hence an amelioration in the bending moment. The specimens failed because of the simultaneous effect of bending moment and axial compression. The ability of specimens to withstand bending is reduced as axial compression is ameliorated. The greatest parallel deformation was reduced as the compression load proportion ameliorated [47]. The STT ratio had a lower effect on the specimens’ peak lateral deformation. Nonetheless, increasing the STT proportion improved longitudinal axial load-carrying strength, bringing the hysteretic functioning of the declining column into alignment and improving hysteretic performance.

### 3.4. Strain Circulation in GLT

**Figure 9:**
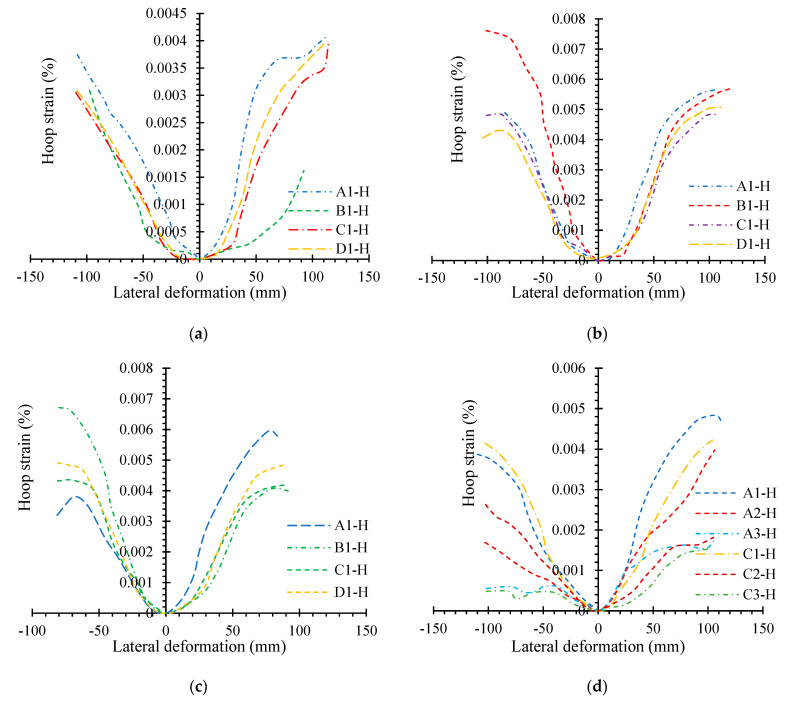
Hoop strains of GLT: (**a**) RAGC100-0.2-STT8 for similar elevation; (**b**) RAGC50-0.2-STT8 for the same elevation; (**c**) RAGC50-0.2-STT10 for diverse elevations; (**d**) RAGC50-0.4-STT8 for the same elevation.

Figure 9 illustrates the glass-FRP hoop strain-horizontal deformation graphs for the specimens. Except for certain approximated strains from fractured strain observations during the load stage, the loop strains of glass-FRP on all of the specimens rose with increasing deformation for the corresponding cross-column of the specimen. When both faces (B and A) were compressed, their strains developed rapidly, but when both faces (B and A) were subjected to tensile stress, their strains developed slowly. The strain in GLT values for sections C and D held consistently. As evidenced by these findings, the GLT used effective confinement to keep RAGC from expanding within the tubes. Face C was strained in the same way as Face D. During the elastic region, both faces (B and A) had symmetrical graphs on the vertical axis, however, the specimens developed fractures and the strain curves became symmetrical. The strain graphs on both faces (B and A) of specimen RAGC50-0.2-STT8 were uneven at the commencement of load in this case, which could be the result of unbalanced concrete infill on face B, prohibiting the FRP regulator from executing its function effectively. The maximum hoop stresses in samples on each of the four faces of specimen RAGC100-0.2-STT8 were higher than in specimens with other RCA substitution rates, possibly indicating its stronger strength and superior distortion functioning. The hoop strains in specimens with higher compression proportions had a straight configuration from yielding deformation to 6δy, suggesting that axial strength can partially prevent the progression of breaks in RAGC, allowing the core concrete to remain in a state of direct flexibility. When the height of specimens was ameliorated, the strains created on face A and side C noticeably decreased.

**Figure 10:**
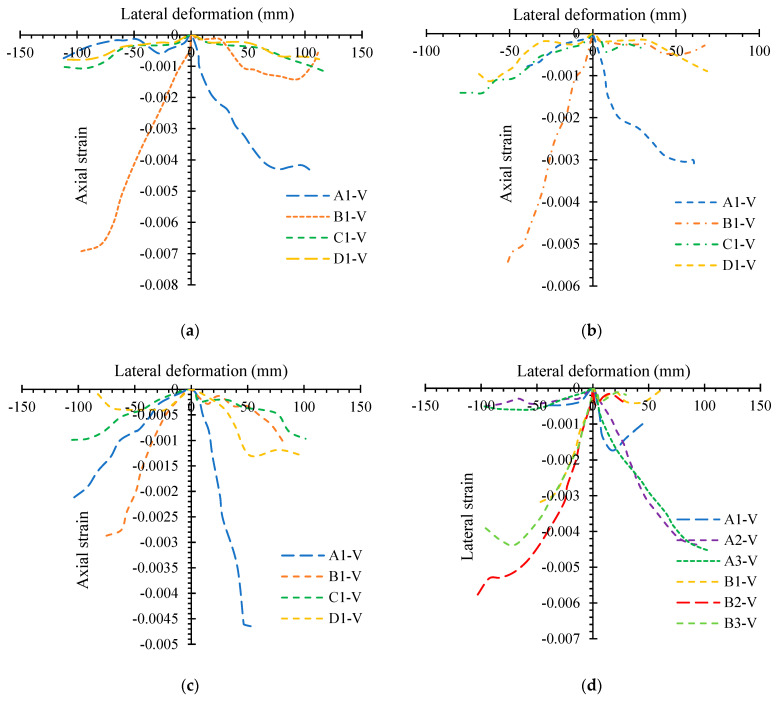
Axial strains of GLT: (**a**) RAGC50-0.4-STT8 for the same elevation; (**b**) RAGC0-0.2-STT8 for the same elevation; (**c**) RAGC100-0.2-STT8 for diverse elevations; (**d**) RAGC50-0.2-STT10 for the same elevation.

Figure 10 shows the axial strains of GLT concerning the specimens’ lateral deformations. The axial stresses of GLT for distinct faces of the specimen exhibited a shifting increment when parallel deformation in the specimen developed. When comparing the compression strains on both sides (B and A) of the specimen with those on separate sides, the compression strains on both sides (B and A) ameliorated at the same rate. The axial stresses of glass-FRP for some specimens decreased rapidly or seemed elastic when the transverse cyclic load was applied, indicating the presence of large cracks in the GLT. When comparing the compression strains on both surfaces (B and A) of the specimen with those on separate sides, the compression strains on both sides (B and A) ameliorated at the same rate. When the lateral cyclic load is transferred to some glass-FRP specimens, the axial stresses reduce quickly or seem elastic, indicating the presence of significant fractures in the GLT. These findings suggested that GLT provided concrete with both keeping and compression stresses. The compression strain on face A of specimen RAGC50-0.2-STT10 ameliorated as the quantity of steel in the specimen ameliorated, while FRP fracture developed on face B later, showing a continuous compression resistance of the STT. The compression strains in the GLT remained extremely small on both faces (B and A), indicating that the STT, rather than the FRP tube, provided the basic tensile commitment. The GLT’s compression face, on the other hand, made a significant dedication to the orientation of compression strength, while the tension face played a minor role. During the advanced load phase, the neutral axis changed to a new point as the specimen was redirected more. Glass-FRP compression strains were comparable during the early load phase as deformation developed at varying heights at the three locations, but they diverged during the late load phase due to the formation of glass-FRP tube fractures. As the sectional area of height dropped, the compression strains ameliorated somewhat, indicating that the GLT could tolerate fractional compression strength.

### 3.5. Ductility

The results of the rotations (R) and ductility (u) of the specimens are shown in Table 5. $P_{k}$, $P_{y}$, and $P_{u}$ are the maximum and damage-parallel yielding compressions, correspondingly. The transverse deformations are k, y, and u when compared to $P_{k}$, $P_{y}$, and $P_{u}$, correspondingly. Rotation and ductility have shown a positive link. The ductility index ranged from 7.1 to 9.7, indicating 2 to 3 times more significance than the required value of three for the seismic elastic zone. A peak range of 0.035–0.059 is also more significant than the required value of 0.020. These findings show that plastic deformation performance is common in FGSTCs.

The ductility of the specimens with shifting RAGC replacement rates decreases somewhat with increasing RCA substitution rates, which could be caused by the RAGC’s material features. The ductility of the specimens generally decreases as the RAGC strength and elastic modulus ameliorate. Ductility ameliorates in proportion to the STT ratio, corresponding to the positive contribution of the STT. The specimen’s ductility was significantly affected by the compression percentage. The RAGC50-0.4-STT8 specimen has 26% less ductility than the RAGC50-0.2-STT8 specimen. The deformation implementation of the specimen declines as the compression area of the specimen due to a larger compression ratio, to the point where the specimen faces unexpected damage due to RAGC smashing in the strength zone.

### 3.6. Cumulative Failure of Specimens

**Figure 11:**
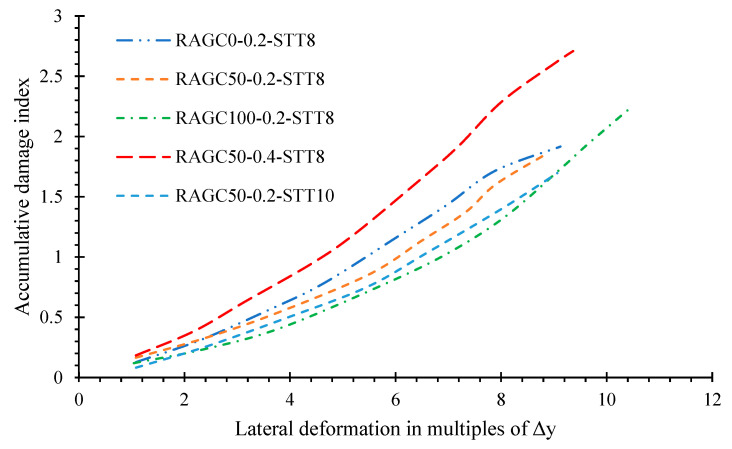
Accumulative damage index.

The cumulative failure of specimens was determined using a two-variables model [58,59] based on energy dissipation and distortion failure enlargements as indicated by the following expression:

(1) $$D=\left(\frac{\delta_{k}}{\delta_{u}}\right)+\beta \left(\frac{\int dE}{P_{y}\delta_{u}}\right)$$

where D is the aggregate value; $\delta_{k}$ and $\delta_{u}$ are the peak deformations of the specimen under seismic behavior and uniaxial load, separately; $\delta_{k}$ equal as 0.62$\delta_{u}$ [60]. Moreover, ∫dE is the total plastic loss of energy of specimen; $P_{y}$ is the load capacity of the specimen at yielding, and β is the loop lateral load coefficient that may be determined with the RAGC replacement level. As seen in Figure 11, the simultaneous failure deformation graphs of each specimen tend to nonlinear turns of events. When the subjected force is in the early stages, the overall failure results of the specimen are minor and ameliorate at a slower rate as the deformation expands. The simultaneous failure of the specimens ameliorates when the load is at a later phase, essentially because the aggregate failure patterns are larger than one, indicating that the specimen failed spectacularly.

When it comes to the influence of the three test parameters, an amelioration in the RCA substitution rate reduces the overall failure of each specimen somewhat. The aggregate failure index of specimen RAGC100-0.2-STT8 is 17% higher than that of specimen RAGC0-0.2-STT8 during the load stage of 8y. The effect of load percentage is so clear that the aggregate failure values of the specimen with a higher compression load proportion (specimen RAGC50-0.4-STT8) are generally higher than that of the sample with a lower compression load proportion (specimen RAGC50-0.2-STT8).

### 3.7. Dissipation of Energy Capacity

**Figure 12:**
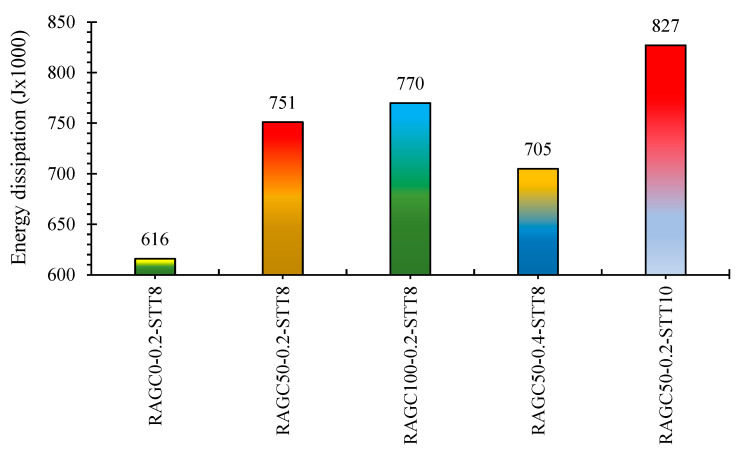
Total dissipation of energy of specimens.

The total energy dissipation of specimens can be used to analyze seismic performance and energy capacity dissipation. In this study, the following relationship is utilized to calculate total energy dissipation:

(2) $$E_{s}=\overset{n}{\underset{i=1}{\sum }}S_{ABCDA}$$

where $i^{th}$ refers to the specimen’s hysteresis loop, and $S_{ABCDA}$ refers to the total portion under that hysteresis loop. The size of each is calculated by using the area of all hysteresis loops is determined using PC programming. Figure 12 shows the absolute energy loss of all five specimens. The energy limit decreases rapidly as the RAGC replacement rate is ameliorated. When compared to a specimen made entirely of NAC the absolute energy spreading of specimens RAGC50-0.2-STT8 and RAGC100-0.2-STT8 are 22% and 25% higher, correspondingly. The specimen’s total energy dispersion is reduced by increasing the compression-load ratio. The energy loss of specimen RAGC50-0.4-STT8 is 7% lower than that of specimen RAGC50-0.2-STT8. The ability to dissipate energy capacity could be improved by increasing the STT ratio. The total energy dissipation of specimen RAGC50-0.2-STT10 is 8.3 × 10^5^ J, which is 10% more than the total energy dissipation of specimen RAGC50-0.2-STT8.

The analogous viscous damping constant ($h_{e}$) and the energy dissipation constant (E) are two separate metrics used to evaluate the seismic resistance functioning of the specimen, as shown in the formulae below [61]:

(3) $$E=S_{A}/\left(S_{B}+S_{C}\right)$$

(4) $$h_{e}=E/2\pi$$

The linkages of the positive highest load on the hysteresis loop, the positive highest deformation on the deformation compression, and the start of orientation define the three-sided region $S_{B}$, where $S_{A}$ is the entire area of a hysteresis loop. Furthermore, $S_{C}$ is a three-sided zone defined by the linkages of the negative highest load on the hysteresis loop, the negative highest deformation on the deformation compression, and the origin of orientation.

**Figure 13:**
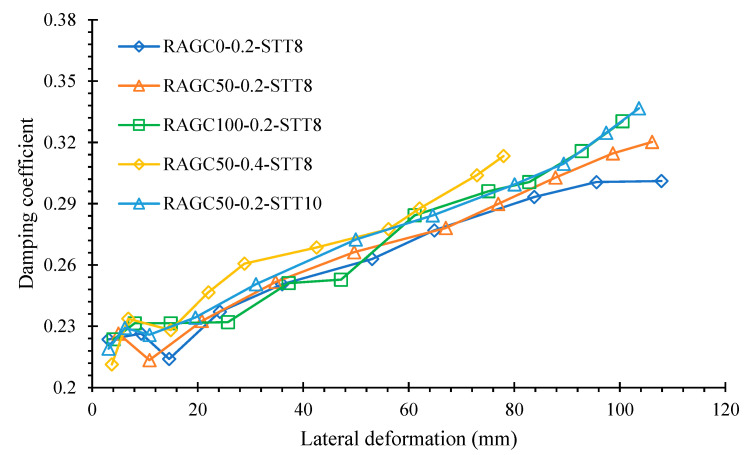
Viscous damping coefficients of specimens.

The viscous damping coefficients against the lateral deformation for the analyzed specimen are shown in Figure 13. Before the initiation of specimen yielding with no specific association with three testing variables, this metric had a value of 0.21. When the specimens begin to yield, this value improves as the deformation of each specimen ameliorates, indicating an amelioration in energy dissipation. When the specimen deformation is minor, the influence of the RAGC replacement rate on the coefficient of damping is very limited. However, when the deformation is larger, especially close to the damage deformation, the impact ameliorates. At the deformation of the deformation, the damping coefficients of specimens RAGC50-0.2-STT8 and RAGC50-0.2-STT10 are 6 percent and 8 percent higher than natural aggregate concrete specimen, correspondingly. The coefficient of the RAGC50-0.2-STT8 specimen was higher (5 percent higher than the coefficient of the RAGC50-0.4-STT8). In general, increasing the RAGC replacement rate and steel ratio, as well as lowering the compression load proportion, will improve energy dissipation. The results show that the damping coefficients for each specimen range between 0.21 and 0.32, which are satisfactory values, as these parameters should be between 0.1 and 0.2 for normal strength concrete columns. These findings show that the FGSTCs specimens have a higher energy dissipation capacity and better earthquake resistance under quasi-static stress.

### 3.8. Stiffness and Axial Capacity

**Figure 14:**
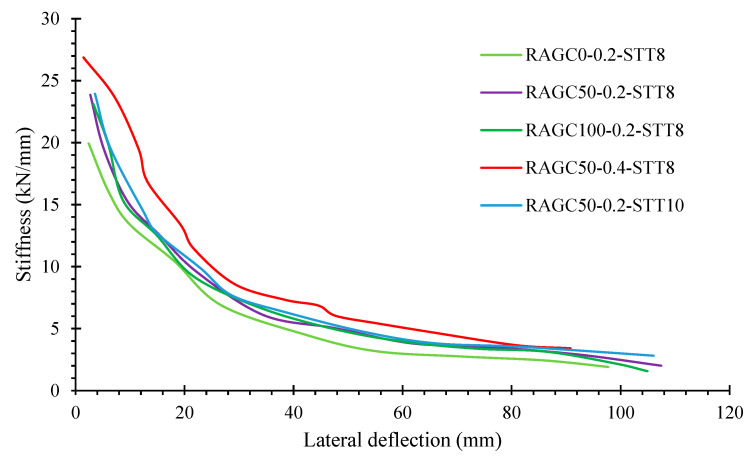
Degradation of stiffness of specimens.

The stiffness functioning of the specimens was evaluated in this investigation using secant stiffness, as stated by the expression:

(5) $$K_{gi}=\left(\left|+P_{ki}\right|+\left|-P_{ki}\right|\right)/\left(\left|+\Delta_{ki}\right|+\left|-\Delta_{ki}\right|\right)$$

where $+P_{ki}$ and $-P_{ki}$ represent the highest loads in the positive and negative directions, correspondingly, during the major pattern of the $i^{th}$ phase of load; and $+\Delta_{ki}$ and $-\Delta_{ki}$ are the highest deformations in the positive and negative ways, individually, during the principal pattern of the $i^{th}$ phase of load application. As demonstrated in Figure 14, the deterioration of stiffness for each specimen may be generated by regulating secant stiffness alongside specimen deformation. The stiffness of the specimen ameliorated slightly as the RAGC replacement rate ameliorated. However, the RAGC replacement level had a minor influence during the deformation. In comparison to the specimen with a lower compression load proportion, the elastic stiffness of the specimen with a higher compression load contribution was more noticeable, but its stiffness decay was also more obvious through the latter load stage. Figure 14 demonstrates that the stiffness-deformation functioning of specimens with various STT ratios was similar, implying that the STT ratios utilized in these studies had only a minimal impact on stiffness reduction. For instance, the stiffness of the five specimens decreased more dramatically during the start of the load period than the late load phase, when it decreased steadily and without surprise.

**Figure 15:**
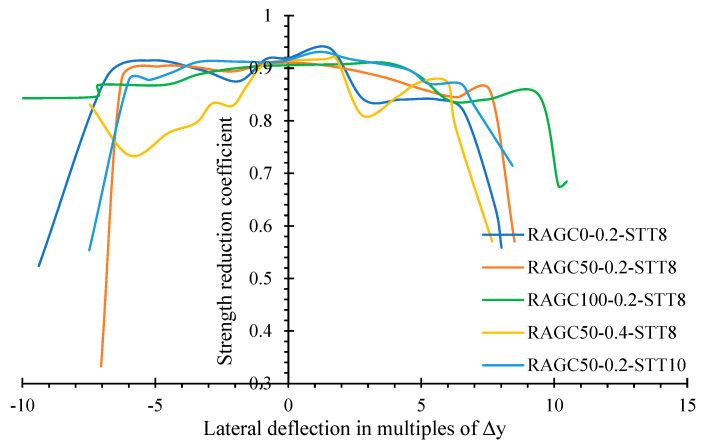
Reduction in load-carrying strength of specimens.

Meanwhile, the axial load capacity of the debasement constant, defined as the percentage of the peak compression of the last major loop of loads, can be used to explain the reduction in axial load-carrying capacity for each specimen under cyclic dumping and reload. Figure 15 depicts the relationship between the axial strength reduction coefficient and the specimen deformation. Because of the yielding and failure of the STT, the decreasing coefficient has a minimal index in the first load phase and then drops dramatically in the final load phase. The axial load-carrying capacity degradation patterns were compared between specimens with varying RCA substitution rates. Even though it provided a higher strength and higher strain of the RAGC, the axial load-carrying capacity debasement of specimen RAGC100-0.2-STT8 with total RAGC replacement was extremely limited. The axial load-carrying capacity loss for the specimen with the higher steel ratio was slightly reduced. In comparison to the influence of other parameters, high strength caused the axial strength to decrease more rapidly, hastening the RAGC and STT damage.

## 4. Conclusions

The goal of this study was to look at the seismic efficiency of five FGSTCs when they were subjected to both cyclic and axial compression loads. In comparison to double-skin tube confined columns with natural aggregates, the simultaneous impact of internal STT and exterior GLT resulted in lower degradation in the seismic execution of created specimens. Moreover, the FRPs and RAGC aggregates in the produced specimens point to long-term sustainability. The following are the primary conclusions that can be drawn from this research:

1. The horizontal load capacity of FGSTCs was 16.9% greater than that of equivalents having natural aggregate. The energy loss of the FGSTCs was 24.5% higher than that of their natural aggregate equivalents. As a result, FGSTCs have a higher axial load-carrying capacity and greater energy loss because of these characteristics. FGSTCs may have a 16.9% lower cumulative failure rate than their natural aggregate counterparts.
2. The FGSTCs had rounded hysteretic loops, and their skeleton graphs showed a significant ameliorate in horizontal deformations before the specimens reached their greatest parallel loads. The FGSTCs’ ductility levels ameliorated from 7.1 to 9.7, and their maximum rotations ameliorated from 0.035 to 0.059, which were two to three times higher than the required value for seismic structural specimens, which should be 0.020. The above findings indicate a common seismic appearance of FGSTCs that is controlled by the exterior GLT and the inward STT.
3. As the curvature ameliorated, the RAGC replacement proportion, accumulative failure, and ductility of FGSTCs all decreased somewhat, whereas energy dissipation and elastic stiffness ameliorated slightly. The axial load-carrying capacity of the specimen did not affect the RCA substitution rate. Ductility, curvature, accumulative failure, and energy dissipation were all commonly affected when the compression load proportion was ameliorated. The steel ratio, rather than the axial load-carrying capacity, played a larger role in widening the transverse deformation of the FGSTCs.
4. The hoop and axial strains of FRP enhanced most rapidly in the compression zones of the specimens and were reduced by increasing the specimen height. Before beginning the FRP cracking process, the hoop and axial stresses versus lateral deformation graphs at comparable column heights on the compressive face and tensile face would be symmetric on the ascending compressive face. The compressive face of the GLT in an FGSTC produced an insufficient engagement to carry the axial capacity while the tension face demonstrated limited engagement; as a result, the main responsibility of the STT in an FGSTC was to handle the tensile loads. In seismic zones, this investigation recommends the adoption of an efficient, lightweight, sustainable, and durable double-skin-tubed compression member.

## Figures and Tables

**Table 1:** Properties of RCA.

Parameter	Value	Parameter	Value
Specific gravity	2.22	Apparent density	2587 kg/m^3^
Los Angeles abrasion	44.5%	Bulk density	1238 kg/m^3^
Water absorption at 24 h	6.7%	Minimum size	4.75 mm
10% fine value	137	Maximum size	12 mm

**Table 2:** Mix proportions for RAGC (kg/m^3^).

Ingredient	RAGC-0	RAGC-50	RAGC-100
Water	126	126	126
Sand	650	650	650
Natural coarse aggregates	1168	584	0
RCA	0	392	784
Superplasticizer	20	20	20
Fly ash	245	245	245
GGBS	166	166	166
Na_2_SiO_3_	108	108	108
NaOH solution	39	39	39

**Table 3:** Parameters of STT.

Sr. No.	$D_{st}$ (mm)	$f_{y}$ (MPa)	$t_{s}$ (mm)	$f_{u}$ (MPa)	$\varepsilon_{y}$ (%)	$E_{s}$ (GPa)	φ (%)
1	300	318	8	482	0.181	210	26.1
2	300	366	10	465	0.237	210	22.3

**Table 4:** Test program.

Sample ID	r (%)	ρ (%)	n	$t_{s}$ (mm)	$D_{s}/t_{s}$	$f_{cu}$ (MPa)	$f_{c}$ (MPa)	$\varepsilon_{u}$ (%)
RAGC0-0.2-STT8	0	3.04	0.2	8	37.5	36.9	32.5	0.265
RAGC50-0.2-STT8	50	3.04	0.2	8	37.5	33.8	30.7	0.285
RAGC100-0.2-STT8	100	3.04	0.2	8	37.5	32.2	28.9	0.318
RAGC50-0.2-STT10	50	3.81	0.2	10	30.0	36.8	31.4	0.278
RAGC50-0.4-STT8	50	3.04	0.4	8	37.5	35.7	34.6	0.332

**Table 5:** Measurement of rotations and ductility.

Specimen	$P_{k}$ (kN)	$\Delta_{k}$ (mm)	$P_{y}$ (kN)	$\Delta_{y}$ (mm)	$P_{u}$ (kN)	$\Delta_{u}$ (mm)	$u=\Delta_{u}/\Delta_{y}$	$\theta =\Delta_{u}/H$
RAGC0-0.2-STT8	214	37	141	9	187	84	9.3	0.051
	−236	−86	−136	−10	−233	−98	9.8	0.059
RAGC50-0.2-STT8	220	33	149	8	187	71	8.9	0.043
	−284	−96	−172	−11	−284	−96	8.7	0.058
RAGC100-0.2-STT8	250	38	163	10	223	74	7.4	0.045
	−206	−59	−126	−8	−204	−74	9.3	0.045
RAGC50-0.4-STT8	288	19	234	8	245	57	7.1	0.035
	−264	−47	−192	−9	−240	−65	7.2	0.039
RAGC50-0.2-STT10	249	69	159	9	236	87	9.7	0.053
	−279	−77	−161	−10	−277	−87	8.7	0.053

## Data Availability

All data have been presented in this paper.
